# Handheld Fluorescence Spectrometer Enabling Sensitive Aflatoxin Detection in Maize

**DOI:** 10.3390/toxins15060361

**Published:** 2023-05-27

**Authors:** Lien Smeesters, Thomas Kuntzel, Hugo Thienpont, Ludovic Guilbert

**Affiliations:** 1 Department of Applied Physics and Photonics, Brussels Photonics (B-PHOT), Vrije Universiteit Brussel and Flanders Make, Pleinlaan 2, 1050 Brussels, Belgium; 2 GoyaLab, Institut d’Optique d’Aquitaine, Rue François Mitterrand, 33400 Talence, France

**Keywords:** aflatoxin, fluorescence, handheld spectrometer, spectroscopy, food safety, optical sensing, maize

## Abstract

Aflatoxins are among the main carcinogens threatening food and feed safety while imposing major detection challenges to the agrifood industry. Today, aflatoxins are typically detected using destructive and sample-based chemical analysis that are not optimally suited to sense their local presence in the food chain. Therefore, we pursued the development of a non-destructive optical sensing technique based on fluorescence spectroscopy. We present a novel compact fluorescence sensing unit, comprising both ultraviolet excitation and fluorescence detection in a single handheld device. First, the sensing unit was benchmarked against a validated research-grade fluorescence setup and demonstrated high sensitivity by spectrally separating contaminated maize powder samples with aflatoxin concentrations of 6.6 µg/kg and 11.6 µg/kg. Next, we successfully classified a batch of naturally contaminated maize kernels within three subsamples showing a total aflatoxin concentration of 0 µg/kg, 0.6 µg/kg and 1647.8 µg/kg. Consequently, our novel sensing methodology presents good sensitivity and high potential for integration along the food chain, paving the way toward improved food safety.

## 1. Introduction

Food traceability and quality control of toxins are major challenges for the European Union states and citizens [1]. The presence of aflatoxins in food and feed products is considered one of the most important food safety issues in the world. Aflatoxins are produced by the fungi *Aspergillus flavus* and *Aspergillus parasiticus.* Several types of aflatoxins occur in nature, of which aflatoxin B1 is the most dominant and toxic one, related to serious health risks like liver diseases and cancer [2]. Moreover, aflatoxins can contaminate food both before and after harvest, and they cannot be identified using visible inspection and cannot be destroyed by any form of food processing [3]. Aflatoxins have been detected in a wide range of food products, including maize, pistachios, fruits, almonds and peanuts. However, as a case study, we focus on aflatoxin contamination of maize, because maize is a staple food in many countries and is cultivated in climates that show an extensive presence of fungi [2,4,5], giving rise to permanent high aflatoxin contamination levels as also reported by the DSM World Mycotoxin Survey 2022 [6] (Table 1). In addition, the European Food Safety Agency (EFSA) is expecting further increases in contamination levels due to climate change [7].

The European Commission sets maximum levels of aflatoxins in food and feed products to protect human and animal health. Considering maize to be subjected to sorting or other physical treatments before human consumption, or to be used as an ingredient in food, the maximum allowed total aflatoxin concentration, i.e., the sum of aflatoxin B1, B2, G1 and G2, is set to 10 µg/kg. The maximum allowed aflatoxin B1 concentration is furthermore limited to 5 µg/kg [1]. High importance is given to aflatoxin B1, as this is the most toxic aflatoxin. Different regulations apply throughout the world. Depending on the country and respective authority, different limits are set for different food and feed products, and whether considering the total amount of aflatoxins and/or the presence of aflatoxin B1 [8,9,10,11]. The Unites States of America limits the total amount of aflatoxins to 20 µg/kg, for all types of food, except milk. Brazil limits the total amount of aflatoxins in maize to 20 µg/kg, while China sets a limit of 20 µg/kg for aflatoxin B1. Japan enforces for all food products a limit on the total aflatoxin concentration of 10 µg/kg. Indonesia places a limit of 15 µg/kg for aflatoxin B1 in maize and 20 µg/kg for the total amount of aflatoxins.

To verify and comply with regulations, aflatoxins are typically detected using chemical analysis, including high-performance liquid chromatography (HPLC), liquid chromatography–tandem mass spectrometry (LC-MS/MS) and enzyme-linked immunosorbent assays (ELISA). A comparison of the different analytical methods for aflatoxin detection was previously published by Wacoo et al. [12] and Miklós et al. [10], while Jallow et al. provided an extensive summary on aflatoxin detection methods including both chromatographic-based methods, immunochemical methods and spectrometric-based methods [8]. The chromatographic methods show excellent precision, selectivity and low limits of detection (below 1 µg/kg), while enabling the simultaneous screening of multiple mycotoxins, but requiring high-end bulky equipment. The immunometric assays are available in rapid tests kits allowing a fast and easy-to-use analysis, but generally with a slightly larger limit of detection. As their main drawback, the analytical methods are sample-based and destructive, while also requiring an extensive sample preparation involving the extraction of the aflatoxin from the matrix [13]. Due to the localized presence of the toxins in food products and crops, analyses based on bulk sample testing often give a limited view of the degree of contamination, inducing a large amount of food waste, without entirely preventing the toxins from entering the food chain [3]. Consequently, to increase food safety, we pursued a non-destructive, reagent-free, cost-efficient and accurate aflatoxin detection using optical spectroscopy that can be applied to individual maize kernels.

Optical spectroscopy has been identified as a promising sensing technique for aflatoxin detection in food [14,15,16]. Particularly, different spectroscopic sensing techniques have already been investigated, including fluorescence spectroscopy, Raman spectroscopy and reflection spectroscopy, covering the visible, near-infrared (NIR) and short-wave infrared (SWIR) spectral bands. Considering fluorescence spectroscopy, ultraviolet excitation is typically used, while the fluorescence signal is detected within the visible wavelength range. Considering reflection spectroscopy and hyperspectral imaging, interesting spectral bands at 700–970 nm, 1146 nm, 1432 nm, 1475 nm, 1510 nm, 1729 nm, 2274 nm and 2344 nm have been previously identified [17]. An overview of the latest contributions focusing on optical sensing of aflatoxins in maize is given in Table 2. Most of these references were published in 2021–2022, indicating the current high importance of this research field. Different classification performances have been reported, influenced by the sample type (ground samples, bulk, or single-kernel analysis) and the considered contamination levels. Most of the measurement configurations are based on research-grade laboratory setups. In addition, an increasing use of machine-learning algorithms for spectral data processing can be observed. Current research generally targets lowering the limits of detection, while improving selectivity and miniaturizing the design.

We pursued the development of a miniaturized handheld aflatoxin-fluorescence sensing unit, enabling the non-destructive sensing of individual maize kernels and allowing the identification of localized contamination within a product batch, while exceeding the state-of-the-art sensing units. We previously demonstrated aflatoxin sensing in maize kernels using a research-grade laboratory setup enabling the differentiation between maize kernels containing 0 µg/kg and 72 µg/kg of aflatoxins [18]. Our current research targets the optimization and miniaturization of this research-grade setup towards a handheld design that can be applied along the whole food chain, while tackling the natural variation and enabling sensitive detection complying with the European legislation. This includes optimization of the illumination and detection optical path, together with the development of a novel sensing algorithm to classify the samples.

This paper specifically presents the development of a novel, non-destructive aflatoxin-sensing methodology using a handheld fluorescence spectrometer, including the optimization of the spectroscopy measurements, spectral data processing and spectral feature selection. A two-fold performance evaluation of the handheld fluorescence sensing unit was made considering contaminated certified maize powder, as well as individual maize kernels, and by evaluating the classification performance using chemical analysis. We believe our novel handheld fluorescence spectrometer contributes to the state-of-the-art by offering an accurate detection within a compact unit that enables an on-site analysis.

## 2. Optical Fluorescence Spectroscopy Results

Fluorescence spectroscopy measurements were performed considering two types of samples: (1) homogeneous certified maize powder (Section 2.1) and (2) naturally contaminated maize kernels (Section 2.2). The homogeneous maize powder was used for benchmarking of the handheld IndiGo spectrometer, with respect to the research-grade laboratory setup. The naturally contaminated maize kernels offer validation for inhomogeneous contaminated samples. All measurements were performed using an excitation wavelength of 365 nm. This excitation wavelength was selected because of the high aflatoxin absorbance, in combination with the low absorbance of fluorescent proteins. We prefer an excitation wavelength that is strongly absorbed by the aflatoxins, since an increasing amount of absorbed photons gives rise to an increasing amount of excited electrons and thus also to an enlarged number of fluorescent photons [29]. This was also previously validated by Ghalkhani et al., who studied the influence of excitation wavelength on aflatoxin B1 fluorescence in acetonitrile, and observed an increasing fluorescence emission when increasing the excitation wavelength from 240 nm to 340 nm and 360 nm [30]. In addition, the intrinsic fluorescence of the food products needs to be taken into account. Maize contains the fluorescent proteins tryptophan (Trp), tyrosine (Tyr) and phenylalanine (Phe), which all show a strong absorbance in the 200–300 nm range [31]. Consequently, we avoided using excitation wavelengths within this range, since otherwise the fluorescence of these proteins would influence our measurements. Finally, the use of 365 nm as the excitation wavelength for aflatoxin detection in maize has previously shown promising results, and significantly better results than when using 405 nm laser light [18].

### 2.1. Fluorescence of Certified Aflatoxin-Contaminated Maize Powder

**Figure 1:**
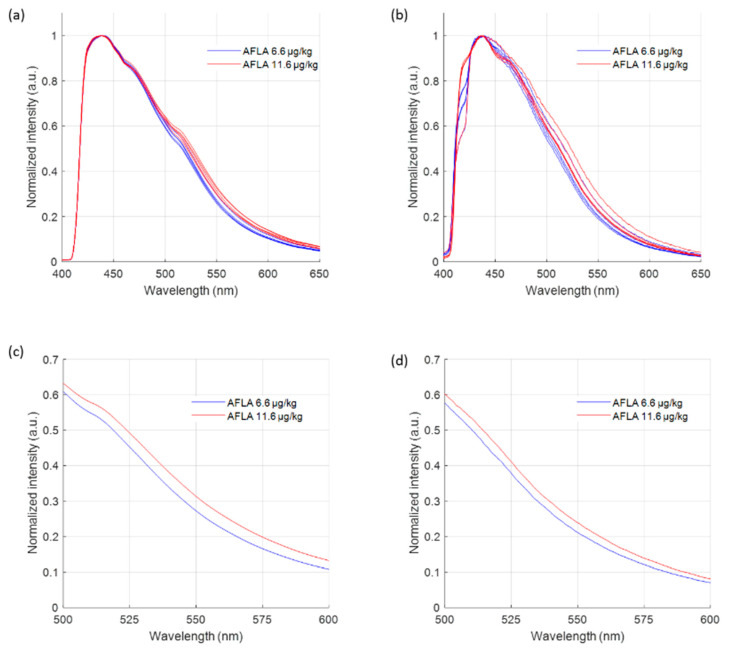
Fluorescence spectra of the certified maize powder, indicating an accurate detection and successful miniaturization. Measurements using (**a**) the research-grade setup and (**b**) the handheld unit. A close-up of the 500–600 nm wavelength range of the mean fluorescence spectra shows a clear wavelength shift for both (**c**) the research-grade setup and (**d**) the handheld unit.

The low (6.6 µg/kg aflatoxin) and medium (11.6 µg/kg aflatoxin) contaminated maize powder both show a fluorescence signal due to the natural fluorescence of maize (Figure 1). In agreement with previous published research, a wavelength shift of the fluorescence spectrum is observed with increasing aflatoxin contamination [25,32], which is especially visible between 500 nm and 600 nm. The present aflatoxin binds to the different natural constituents in the maize, influencing its natural fluorescence. Specifically, the mean fluorescence spectrum using the high-end research-grade setup shows a fluorescence emission maximum at 439 nm for the low contaminated sample and at 438 nm for the medium contaminated sample. The mean fluorescence spectrum using the handheld measurement unit shows a maximum at 437 nm for both the low and medium contaminated maize powder. The small difference in the fluorescence maximum is less expressed when using the handheld module, due to the slightly larger measurement variation and measurement error than when using the research-grade setup. When comparing the mean fluorescence emission shift between the low and medium contaminated sample at a normalized intensity of 0.3, a wavelength shift of 6.93 nm is observed when using the research-grade setup, while when using the handheld device, a wavelength shift of 4.33 nm can be observed.

Both the research-grade setup and the handheld unit enable differentiation between the low and medium contaminated sample, without the need for advanced chemometrics or machine learning, thanks to the highly accurate measurement configurations (Figure 1c,d). The novel handheld unit shows high performance, close to that of the research-grade setup, indicating a successful miniaturization of the latter bulky laboratory setup. Furthermore, since the medium contaminated sample slightly exceeds the European limit of 10 µg/kg, while the low contaminated one features a lower concentration, we can also confirm that our sensitivity is sufficient to comply with the regulations.

### 2.2. Fluorescence Emission of Naturally Contaminated Maize Kernels

We pursued using the handheld fluorescence measurement unit to sort a batch of aflatoxin-contaminated maize kernels, with a priori unknown contamination, within three subsamples featuring different contamination levels. We used the following three-step approach: (1) measurement of the fluorescence spectra of the individual maize kernels; (2) interpretation of the fluorescence spectra and development of an identification algorithm enabling us to classify the maize batch into three subclasses, each with a different contamination level, while also focusing on the lowest contamination levels and the limit of detection; (3) classification of the maize kernels within the three defined subclasses and validation of the sorting algorithm using chemical analysis.

**Figure 2:**
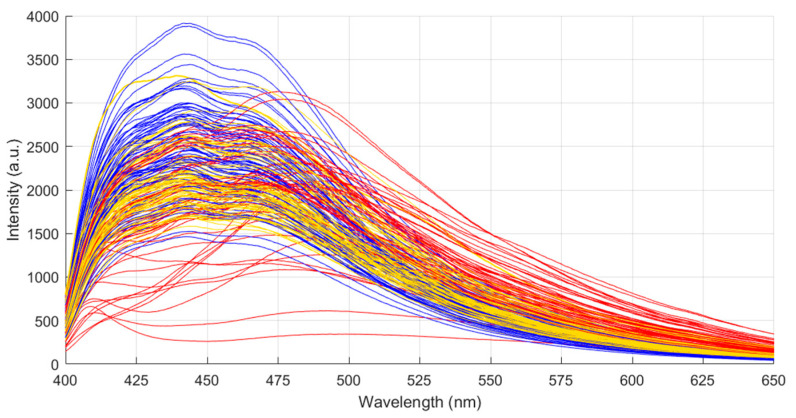
Fluorescence spectra of maize kernels indicating a wavelength shift with aflatoxin contamination. The colors (blue, yellow, red) correspond to the subsequent subdivision of the batch.

In the first step, the fluorescence spectra of 100 individual maize kernels were measured using the handheld IndiGo fluorescence unit. Each kernel was measured twice (once on each side), for which all fluorescence spectra are presented in Figure 2. Similar to the measurements of the maize powder described in Section 2.1, the fluorescence emission wavelength shows a varying shift within the sample batch, indicating the presence of different aflatoxin concentrations.

In comparison to the fluorescence spectra of the homogeneous maize powders, the wavelength shift of the fluorescence emission maximum is more largely pronounced, indicating larger contamination differences. Particularly, the maximum fluorescence emission wavelength varies between 438 nm and 493 nm. In addition, a larger variation within the spectral data is observed due to the natural variation. This is in agreement with previous research that showed a skewed aflatoxin distribution in bulk maize batches [33,34]. The majority of kernels typically feature a low aflatoxin contamination, while there are a few hotspots where the kernels are highly contaminated.

**Figure 3:**
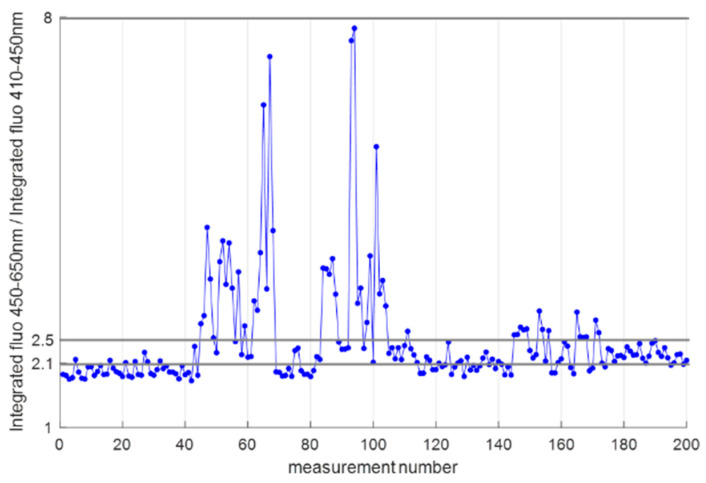
Ratio of the integrated fluorescence intensities for each of the measurements, on which the sample classification is based.

Using these fluorescence spectra, in-depth data processing using MATLAB^®^ was performed in the second step, targeting a sample classification based on the spectral characteristics. To subdivide the samples according to their contamination level, the ratio of the integrated fluorescence spectrum between 450 nm and 650 nm, and the integrated fluorescence spectrum between 410 nm and 450 nm is considered. Preference is given to a ratio of integrated fluorescence signals, since this enables cancelling offsets while resulting in a more robust classification than when only considering the emission maximum. In general, ratios between 1.8 and 8 are observed (Figure 3), where the lowest values for the ratio are expected to correspond with the lowest contamination levels, since the ratio is affected by the wavelength of the fluorescence emission maximum. Based on the given value for this ratio, the measured kernels are subdivided in three groups: (class A) ratio < 2.1, (class B) 2.1 ≤ ratio ≤ 2.5 and (class C) ratio > 2.5. For each maize kernel, two values for the ratio were obtained, corresponding to the two measurements/sample. The highest value for the ratio determined the subgroup since this corresponds to a higher contamination level. Our selection resulted in a classification into a healthy, a low contaminated and a high contaminated class. The thresholds for class A and B are therefore close to each other, since in view of food safety, we also aimed to demonstrate the ability to identify small differences. For each class, it was taken into account that a minimum of 10 g of kernels was required for the chemical analysis.

**Figure 4:**
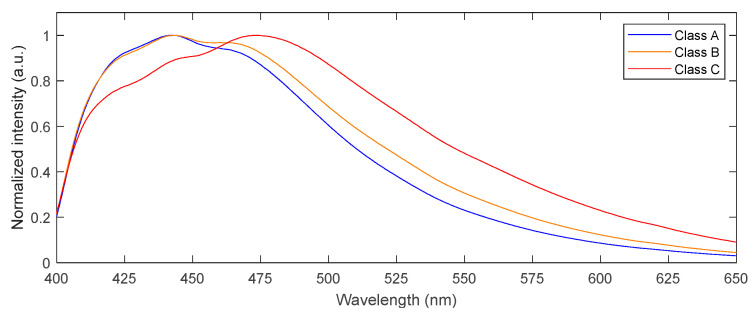
Mean fluorescence spectrum of each of the 3 classes indicating a wavelength shift from class A to B and C.

A wavelength shift can be observed between the mean fluorescence emission spectra of each of the three resulting classes (Figure 4). The fluorescence maximum of class A occurs at a wavelength of 442 nm, while the maximum of class B and C occur at 444 nm and 473 nm, respectively. Furthermore, it can be observed that each of the three mean spectra show two local emission maxima. Class A and class B show the highest intensity at the first maximum, while for class C, the second maximum shows the highest intensity. Comparing class A with class B, the second local maximum of class B shows a higher intensity than that of class A. Consequently, the fluorescence energy distribution within each of the two local maxima is influenced by the presence of aflatoxin, while an increasing fluorescence intensity is observed at larger wavelengths when going from class A to class B and class C. Considering a normalized intensity equal to 0.6, a wavelength shift of 9 nm is observed between class A and class B, while a shift of 24 nm is present between class B and class C.

As a third step, the three subgroups were analyzed by Sciensano, the Belgian National Reference Laboratory for Mycotoxins, Plant Toxins and Marine Biotoxins, using multi-mycotoxin LC-MS/MS. The measured aflatoxin contamination for each of the subclasses is presented in Table 3, indicating a successful classification. Class A shows no aflatoxin presence, while class B shows only a minor aflatoxin B1 contamination, and class C shows high concentrations for both aflatoxin B1 and B2. In contrast to our spectroscopy measurements, the chemical analysis enables us to differentiate between the different types of aflatoxin, while our fluorescent measurement module measures the total aflatoxin contamination. However, our fluorescence measurements allow us to consider individual kernels, while the chemical analysis measures the mean contamination of the whole subgroup, lowering the influence of outliers.

Class A and B only show a difference of 0.6 µg/kg aflatoxin, while a clear differentiation is present between the ratio of the classes (Figure 3) and within their spectral characteristics (Figure 4), indicating that our technology offers a promising sensitivity. Class C shows a strong aflatoxin contamination, corresponding to the high integrated fluorescence ratios. Considering this latter class, particularly the samples with ratios close to 8 greatly increase the contamination level.

## 3. Discussion

Research on the detection, reduction and removal of aflatoxins is indispensable in view of human and animal health. Fluorescence spectroscopy offers a high potential as a rapid, sensitive and specific technique. Aflatoxin sensing using fluorescence spectroscopy has previously shown excellent results, indicated by a 95.7% sensing performance with a cut-off of 10 µg/kg [27], but this method used ground maize samples, requiring advanced data processing, such as quadratic discriminant analysis, and a high-end laboratory setup. We pursued boosting state-of-the aflatoxin sensing by proposing a novel aflatoxin-sensing fluorescence unit, addressing the following four points:

(1) Miniaturization of the measurement unit, enabling the transition from a research-grade setup to a handheld unit.
(2) Measurement of individual maize kernels, without the need for sample preparation, thus addressing the natural variation and inhomogeneity of the samples.
(3) Minimization of the complexity of data processing, allowing fast processing and in-line integration.
(4) Excellent sensitivity, enabling compliance with European legislation.

The proposed handheld fluorescence sensing unit was first benchmarked with respect to a research-grade laboratory setup, indicating successful miniaturization. Following, we present promising sensitivity by demonstrating the differentiation between naturally contaminated maize samples. First, the differentiation between homogeneous maize powder featuring 6.6 µg/kg and 11.6 µg/kg of total aflatoxins was demonstrated. Second, successful sorting of a contaminated maize batch was achieved, with subclasses featuring total aflatoxin concentrations equal to 0 µg/kg, 0.6 µg/kg and 1647.8 µg/kg. This classification shows high sensitivity, enabling the differentiation between contamination differences of only 0.6 µg/kg, while being based on the spectral intensity characteristics of individual kernels, and taking the natural variation into account. This excellent sensitivity indicates promising performance for future integration. We believe the compact aflatoxin sensing unit offers high potential for integration along the whole food chain. Particularly, the handheld device can be used for on-site analysis at the farm, during storage or at the food-processing facility. Furthermore, its use might serve different purposes, from providing a general indication of aflatoxin presence, to enabling more accurate sampling for chemical analysis, while also offering a fast screening tool for the competent authorities. As such, aflatoxin can be identified early in the production process, enabling fast assessment and avoiding cross-contamination. In addition, since the detection is non-destructive and can be applied to individual kernels, an entire batch can be screened while only removing the contaminated kernels, thus increasing food safety while minimizing the amount of food waste.

Future work includes the validation of the technology on a wide variety of maize batches, while extending the natural variation by covering different subtypes and fields and by considering different harvests. Finally, further improvements might be realized by implementing machine learning in the data processing.

## 4. Conclusions

We present a non-destructive aflatoxin-sensing method for individual maize kernels by using a handheld fluorescence unit, enabling rapid evaluation of localized contamination while complying with European legislation. Specifically, a high sensitivity is demonstrated, enabling the sensing of a minimal aflatoxin contamination difference of only 0.6 µg/kg, while considering integrated fluorescence intensity ratios within the data processing. The combination of the high sensitivity and the compact design, together with non-destructive measurement of individual kernels, exceeds state-of-the-art sensing and performance. Furthermore, using the handheld unit, we demonstrated successful classification of aflatoxin-contaminated maize with a similar accuracy as when using a bulky research-grade fluorescence laboratory setup. We therefore believe our presented aflatoxin sensing methodology offers great potential to enhance food safety, while being applicable along the whole food chain thanks to the compact design.

## 5. Materials and Methods

### 5.1. Maize Samples

**Figure 5:**
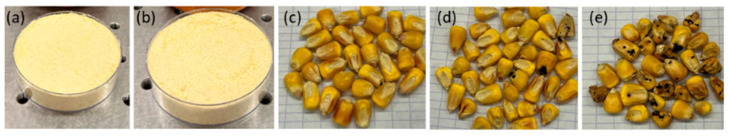
Overview of the considered samples: (**a**) low-level contaminated maize powder; (**b**) medium-level contaminated maize powder; (**c**) maize subsample—class A; (**d**) maize subsample—class B; (**e**) maize subsample—class C.

Two types of samples were considered: (1) certified maize calibration samples (Romer Labs, Butzbach, Germany) and (2) naturally contaminated maize kernels (Figure 5). For the certified calibration samples, we used a low-level and a medium-level ‘Quality Check Aflatoxins in maize’ powder sample, both of which are Biopure Quality Control Materials and feature a total aflatoxin contamination of 6.6 µg/kg and 11.6 µg/kg, respectively. These are naturally contaminated samples that are characterized by Romer Labs using ISO 17025 accredited LC-MS/MS [35], and their stability and homogeneity is assured. The samples were stored between −18 °C and −22 °C. The precise contamination composition of the samples is presented in Table 4.

The naturally contaminated maize kernels originated from Croatia and were divided into 3 subsamples (class A, class B, class C) using fluorescence spectroscopy (Section 2). No sample preparation was applied (Figure 5). Therefore, these naturally contaminated kernels offer a validation for an inhomogeneous contaminated maize batch.

### 5.2. Instrumentation

Measurements were performed using a research-grade fluorescence laboratory setup and handheld IndiGo fluorescence spectrometer. For both measurement configurations, an excitation wavelength of 365 nm was used, corresponding to the aflatoxin absorption characteristics [33]. Particularly, an excitation wavelength with a strong aflatoxin absorbance was used, since the influence of the aflatoxin fluorescence emission onto the natural fluorescence spectrum of the food products can then be maximized.

#### 5.2.1. Research-Grade Fluorescence Measurement Setup

**Figure 6:**
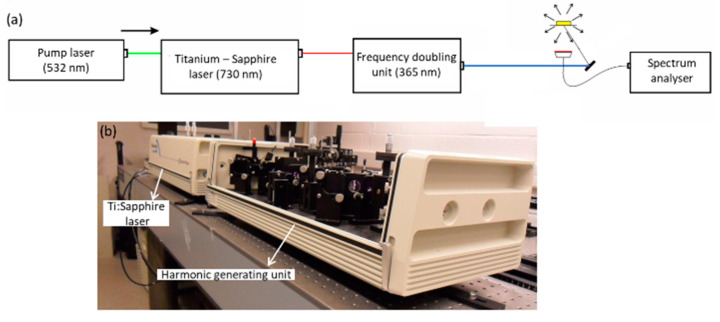
Research-grade laboratory setup: (**a**) schematic representation of the setup, (**b**) photo of the used laser module.

The research-grade laboratory setup comprises a separated excitation and detection path, and has been previously validated for aflatoxin fluorescence characterization [18,19,32]. The sample is excited by illumination with 365 nm laser light, with an excitation power of 65 mW and an illumination spot diameter of 951 µm (Figure 6). This illumination laser light is generated using a tunable laser setup comprising a frequency-doubled Nd:YAG laser (532 nm laser) followed by a tunable titanium-sapphire laser and second-harmonic-generating unit (Newport Spectra-Physics, Utrecht, The Netherlands). The output of the laser is directed towards the sample by use of a mirror. The fluorescence emission is captured in reflection by a collimating lens, coupled into a UVIR600 broadband optical fiber (Avantes, Apeldoorn, The Netherlands) and guided towards the AvaSpec2048 spectrum analyzer (Avantes, Apeldoorn, The Netherlands). We use a collimating lens in combination with a large fiber core diameter (600 µm) to obtain a total acceptance angle of 4.1° that allows capturing the fluorescence signals of a surface area of 39 mm^2^. In front of the detecting fiber, an optical filter (Semrock 405 nm EdgeBasic filter BLP01-405R-25, IDEX Corporation, Lake Forest, IL, USA) is positioned to suppress the excitation light avoiding saturation of the spectrum analyzer. The used spectrum analyzer measures the spectrum between 300 nm and 1100 nm with a resolution of 8 nm. The measurements are performed in a dark environment to avoid the influence of background light. An integration of 2 ms is set during the measurements of maize powder, which was optimized prior to the measurements maximizing the signal to noise ratio. The same measurement settings were used during the measurement of the low and high contaminated maize powder sample.

#### 5.2.2. Handheld IndiGo Fluorescence Spectrometer

**Figure 7:**
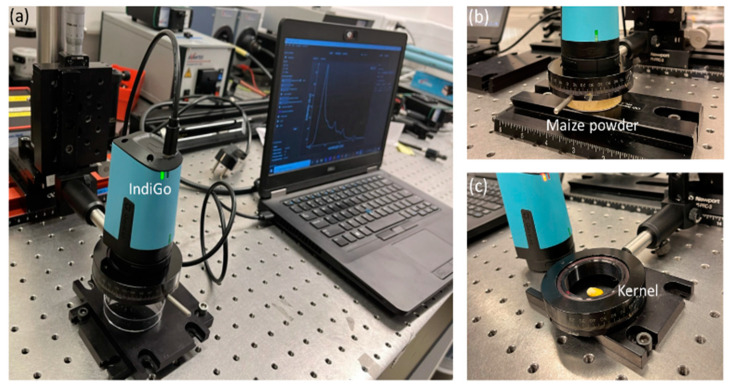
Fluorescence measurement setup using the handheld fluorescence spectroscopy unit: (**a**) IndiGo fluorescence unit with USB connection to a computer, (**b**) positioning of the maize powder samples and (**c**) positioning of the individual maize kernels.

The handheld IndiGo fluorescence spectrometer (GoyaLab, Talence, France) was optimized for aflatoxin sensing, comprising the illumination and detection within the same compact unit featuring dimensions of 76 mm × 45 mm × 53 mm (Figure 7). The illumination optics are composed of a custom LED ring module positioned around the spectrometer entrance slit, comprising 6 high powerful LEDs emitting at 365 nm. In front of the entrance slit, a high-pass filter is positioned, limiting reflections from the excitation light beam to pass to the detector. A spectrometer operating from 400 nm to 720 nm is used, featuring a high-sensitive back-side illuminated sensor. Particularly, the IMX334 CMOS sensor was used, featuring 3840 × 2160 pixels with each a 2 µm pixel size (Sony Corporation, Tokyo, Japan). An integration time of 5 ms was used during the measurements of maize powder, for both the low and high contaminated sample, while an integration time of 175 ms was used during the measurement of maize kernels. Different integration times were considered in view of maximizing the signal to noise ratio. Particularly, a larger integration time was required during the measurements of the maize kernels, because of their smaller sample size, which induced some illumination light losses and thus lower signal levels. A temperature control system was included to ensure the repeatability of the measurements. The spectrometer was attached to a computer using a USB connection, and the SpectroLab software (GoyaLab, Talence, France) was used to capture the spectroscopic data. The IndiGo fluorescence spectroscopy unit was positioned on top of a translation stage, enabling us to position the samples in the center of the entrance slit. Measurements were performed with background light on, mimicking use in the field, but featuring a minimal distance between the sample and the source.

## Figures and Tables

**Table 1:** Worldwide reported aflatoxin contamination in maize in 2022 [6].

	Number of Tested Samples	Percentage of Contaminated Samples	Average Contamination (µg/kg)	Median Contamination (µg/kg)	Maximum Contamination (µg/kg)
Europe	783	11	30	6	370
North America	428	7	47	18	602
South and Central America	3928	18	6	2	565
Asia	983	27	44	20	478
Africa	467	6	45	22	247
Middle East and North Africa	11	45	1	1	3

**Table 2:** State-of-the-art aflatoxin (AFLA) detection using optical spectroscopy.

Spectroscopic Sensing Technique	Sample Type	Measurement Device	Sensing Performance	References
One- and two-photon induced fluorescence spectroscopy	Individual maize kernels	Research-grade laser setup	Classification between 0 µg/kg and 72 µg/kg AFLA	[18,19]
SWIR hyperspectral imaging	Bulk kernels	/	Classification accuracies of 70–96%	[17]
Fluorescence hyperspectral imaging	Bulk kernels	/	Classification accuracy of 87% for 20 µg/kg AFLA	[17]
Fourier-transform NIR + neural network models	Maize powder	Antaris FT-NIR laboratory spectrometer	Root mean square error of prediction = 1.5606 µg/kg for AFLA between 2.5 µg/kg and 41.5 µg/kg	[20]
NIR spectroscopy + support vector machines	Ground samples	Research NIR setup 901.78–1661.24 nm	Root mean square error of prediction = 3.5967 µg/kg for AFLA between 2.6 µg/kg and 61 µg/kg.	[21]
UV-VIS-NIR reflectance + UV-excited fluorescence + random forest model	Single kernel	Custom research LED setup	Accuracy of 95% for 20 µg/kg AFLA	[22]
Raman spectroscopy + support vector machines	Crushed maize	Handheld Raman spectrometer with 785 nm laser	Root mean square error of prediction = 3.5377 µg/kg for AFLA between 2.6 µg/kg and 61 µg/kg.	[23]
NIR spectroscopy + deep learning	Ground samples	Custom NIR spectrometer (901.78–1661.24 nm)	Root mean square error of prediction = 1.3691 µg/kg for AFLA between 2.7 µg/kg and 61 µg/kg	[24]
Fluorescence spectroscopy + multispectral imaging + linear discriminant analysis	Whole kernels	Spectrofluorimeter with xenon lamp + photomultiplier	Classification between 0 µg/kg and 1475 µg/kg AFLA	[25]
NIR hyperspectral imaging + linear discriminant analysis	Individual kernels	In-line laboratory setup using halogen light	Accuracy 88.67–95.56% for AFLA between 0 µg/kg and 100 µg/kg.	[26]
VIS-NIR-SWIR reflectance + machine learning	Ground maize	Camera-based lab setup with imaging spectrograph	82.6–95.7% (cut-off 10 µg/kg)	[27]
Fluorescence spectroscopy + support vector machines	Ground maize	UV LED lab setup with imaging spectrograph	95.7% (cut-off 10 µg/kg)	[27]
Raman spectroscopy + support vector machines	Ground maize	Research lab setup using 785 nm laser + ImSpector spectrograph	87% (cut-off 10 µg/kg)	[27]
UV-VIS-NIR spectrometer + partial least squares	Single kernels	Reflectance research grade setup (304 to 1085 nm)	71, 82 and 92% for 20, 100 and 1000 μg/kg AFLA	[28]

**Table 3:** Results of chemical analysis of the three classified subsamples.

Maize Sample	Aflatoxin B1 (µg/kg)	Aflatoxin B2 (µg/kg)	Aflatoxin G1 (µg/kg)	Aflatoxin G2 (µg/kg)
Class A	0	0	0	0
Class B	0.6	<Limit of quantification	0	0
Class C	1527.1	120.7	0	0

**Table 4:** Aflatoxin contamination of the certified quality control maize powder samples.

Maize Sample	Aflatoxin B1 (µg/kg)	Aflatoxin B2 (µg/kg)	Aflatoxin G1 (µg/kg)	Aflatoxin G2 (µg/kg)
Low level	5.3 ± 2.1	1.3 ± 0.5	<1	<1
Medium level	9.5 ± 3.5	2.1 ± 0.7	<1	<1

## Data Availability

The data presented in this study are available on request from the corresponding author.
